# NF-κB Essential Modulator Deficiency Leading to Disseminated Cutaneous Atypical Mycobacteria

**DOI:** 10.4084/MJHID.2015.010

**Published:** 2015-01-01

**Authors:** Jonathan Braue, Vagishwari Murugesan, Steven Holland, Nishit Patel, Eknath Naik, Jennifer Leiding, Abraham Tareq Yacoub, Carlos N Prieto-Granada, John Norman Greene

**Affiliations:** 1 Morsani College of Medicine, University of South Florida, Tampa, Florida; 2 Division of Infectious Diseases, Moffitt Cancer Center, Tampa, Florida; 3 Laboratory of Clinical Infectious Diseases, National Institute of Allergy and Infectious Diseases, National Institutes of Health, Bethesda, Maryland; 4 Department of Dermatology and Cutaneous Surgery, Morsani College of Medicine, University of South Florida, Tampa, Florida; 5 Division of Infectious Disease and International Medicine, Morsani College of Medicine, University of South Florida, Tampa, Florida; 6 Department of Pediatrics, Division of Allergy, Immunology, and Rheumatology, Morsani College of Medicine, University of South Florida, Tampa, Florida; 7 Division of Infectious Diseases, Moffitt Cancer Center, Tampa Florida; 8 Department of Dermatology, Morsani College of Medicine, University of South Florida, Tampa, Florida; 9 Chief, Division of Infectious Diseases, Moffitt Cancer Centre, Tampa

## Abstract

NF-κB essential modulator (NEMO) is a kinase integral to the macrophage TNF-α pathway, which leads to the intracellular destruction of Mycobacteria species. Defects in the NEMO pathway result in spectrum of diseases, including but not limited to ectodermal dysplasia, Mendelian susceptibility to mycobacterial diseases, and incontinentia pigmenti. In addition, paucity of NEMO can lead to the inability to mount a proper immune response against opportunistic pyogenic and mycobacterial infections, leading to dissemination to various organ systems. This manuscript will discuss the numerous clinical manifestations of NEMO deficiency, the differential diagnosis of atypical mycobacterial infections in immunocompetent adults, and feature a case report of rare isolated susceptibility to disseminated atypical mycobacteria due to a mutation in the first exon of the NEMO gene.

## Introduction

In 1991, Nedorost et al. described two brothers, each affected with rosacea-like lesions discovered to stem from cutaneous infection with *Mycobacterium avium-intracellulare* (MAI).^1^ Neither brother had reason for acquired immunoincompetence, so it was postulated that they potentially harbored a genetic defect in the pathway responsible for the destruction of mycobacterial species.^1^ Though, this defect remained to be uncovered. Then, in 1996 the occurrence of a theoretically X-linked recessive susceptibility to mycobacterial infection was reported^2^. The affected patients were maternally related members of the family revealed to have defective IL-12 production, which is necessary to defend against mycobacteria.^2^ Later that same year, the genetic etiology of Mendelian susceptibility to mycobacterial diseases (MSMD) was first accounted.^3^ MSMD, a congenital syndrome predisposing affected individuals to clinical disease with low virulence non-tuberculous mycobacteria, was first shown to result from germline mutations in the autosomal gene responsible for the production of the IFN-γ receptor ligand-binding chain^3^. Ensuing studies demonstrated five other autosomal genes involved in IL-12-dependent, IFN-γ-mediated immunity resulting in MSMD.^4^ However, the genetic mutation causing the X-linked pattern observed in previous cases of familial mycobacterial infections remained elusive.

NF-κB transcription factor is vital to the innate immune response to numerous pathogens including mycobacteria. Two proteins known as NF-κB inhibitor protein A and B control the immunomodulatory functions of NF-κB. In turn, these proteins are regulated by IκB kinase alpha, beta, and gamma otherwise known as NEMO. The gene encoding NEMO, located on the X chromosome, is activated by TNF-α binding to its receptor leading to downstream destruction of intracellular bacteria via the NF-κB pathway.^5^,^6^ In early 2000, mouse model studies linked NEMO deficiency to the human genodermatosis incontinentia pigmenti.^6^,^7^ Shortly thereafter, novel human mutations in the NEMO gene were shown to lead to the X-linked disorders, ectodermal dysplasia, and incontinentia pigmenti.^8^ Susceptibility to pyogenic and mycobacterial infections were known to be a part of the spectrum of these diseases, and in 2004, Orange et al reported data comprising of seven boys with mutations leading to NEMO immunodeficiency, most of which suffered from mycobacterial illness.^9^ Also, in 2006, a novel mutation in NEMO was reported as a cause of MSMD^6^. Thus, primary immunodeficiency in the NEMO protein contributes further to the differential diagnosis in patients with unexplained susceptibility to atypical mycobacteria.

## Case Report

A 37-year-old Caucasian man presented to our institution for evaluation of several multifocal chronic granulomatous skin lesions. He had a childhood history of asthma and pneumonia, as well as recurrent sinus infections requiring sinus surgery as an adult. Family history revealed a male cousin who was recently hospitalized for a disseminated *Mycobacterium avium-intracellulare* infection involving his liver. The patient denied any recent travel outside the country.

The skin lesions originally erupted seven years prior, starting as a non-painful, non-pruritic plaque on the right shoulder that remained persistent and eventually spread to all four extremities. Sarcoidosis was diagnosed at initial presentation and treated with corticosteroids and hydroxychloroquine. Despite treatment and the initial response, skin lesions remained persistent, and thus subsequent skin biopsies were performed. Results yielded numerous acid-fast bacilli with granulomas. A tentative diagnosis of leprosy was given, and treatment with rifampin, clarithromycin, and minocycline ensued. Unfortunately, this regimen failed to yield any significant degree of improvement. Moreover, taper from the steroid treatment initially given for sarcoidosis was attempted, only to produce worsening of the skin lesions and development of high-grade fevers up to 105.0 ° F requiring hospitalization.

Due to pancytopenia, persistent fevers, and concern for disseminated infection or malignancy, a bone marrow biopsy was performed. Analysis of the biopsy demonstrated granulomas and acid-fast bacilli; culture isolates yielded *Mycobacterium avium-intracellulare* (MAI), rather than the preconceived *Mycobacterium leprae*. Based on these findings, antimicrobial therapy was changed to clarithromycin, ethambutol, and rifampin. Corticosteroids at a dose of 16 mg daily were also continued to prevent rebound exacerbation of the skin lesions.

Despite antibiotic therapy for six months, fevers persisted, and skin lesions advanced with new nodular masses developing quickly over the period of a week. Multiple ulcerations with overlying eschars on the right forearm and torso persisted, and large erythematous nodules with central low-grade ulceration formed on the right lower extremity. In addition, enlarged para-aortic lymph nodes concerning for granulomatous disease were visualized on CT scan.

**Figure 1:**
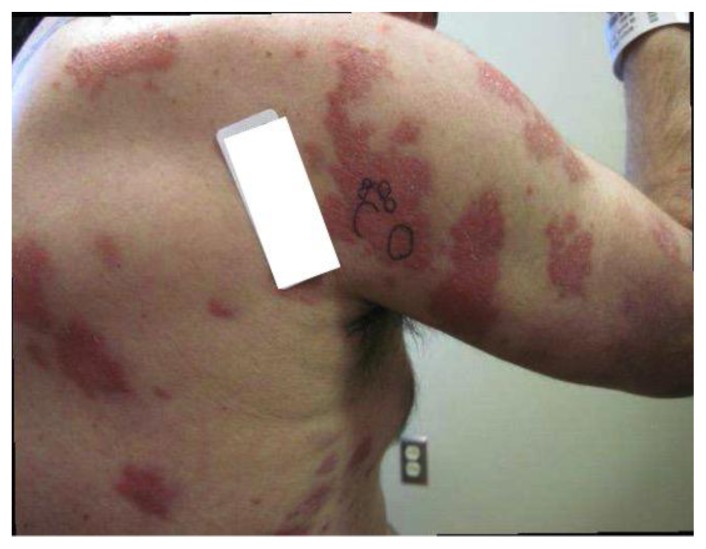


**Figure 2:**
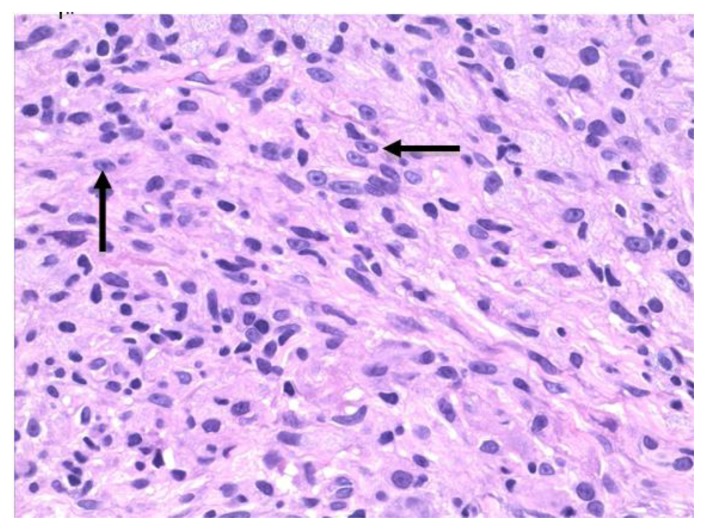


**Figure 3:**
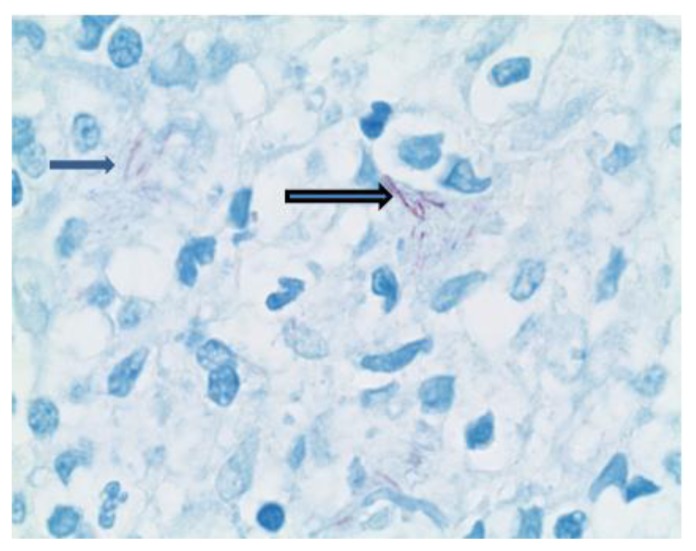


**Figure 4:**
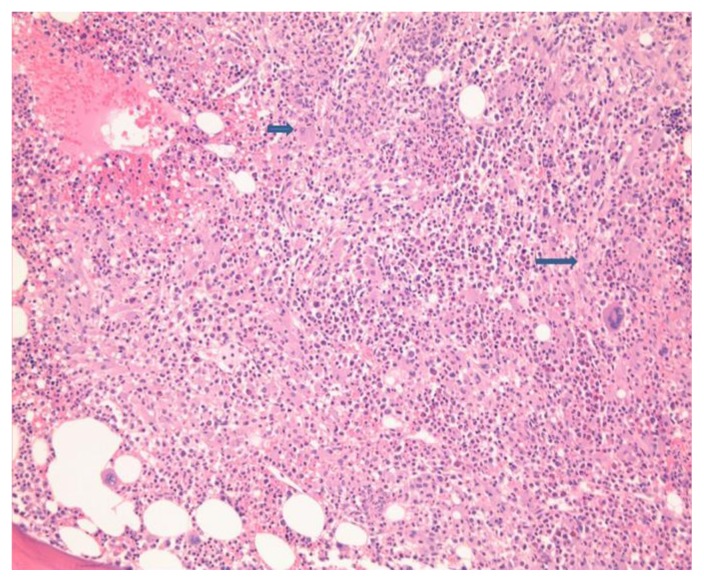


A repeat skin biopsy showed a persistent MAI infection resistant to clarithromycin, ethambutol, and moxifloxacin. Additionally, the pathology report described an atypical T-cell infiltrate in the dermis and positive TCR gene rearrangement raising concern for cutaneous T-cell lymphoma (CTCL). At this time, the patient was referred to us for work-up of his potentially malignant skin condition. At presentation to our institution, lesions consisted of 3 to 4 inches in diameter, diffuse, raised, well-circumscribed erythematous red scaly plaques with a lack of active ulceration (Figure 1). Because of the concern for CTCL, further skin biopsies were performed. Results showed a histiocytic infiltrate admixed with very few small atypical lymphocytes (Figure 2) and long beaded bacilli consistent with mycobacterium, located in the superficial and deep dermis (Figure 3). Isolates demonstrated not only *MAI,* but also the presence of *Mycobacterium simiae*. Bone marrow core biopsy revealed a normocellular bone marrow constituents being populated by foamy histiocytes that are disposed in loosely formed granulomas (Figure 4). The admixed T-cells were clonal and appeared to lack CD7 by flow cytometry, but were positive for CD3, CD4, and CD26. However, the absence of epidermotropism, lymphohistiocytic infiltrate with acid-fast mycobacteria, and ultimately the marked paucity of T-cells and lymphocytes, rendered a clonal lymphoproliferative disorder involving the skin exceedingly unlikely. With CTCL effectively ruled out and compelling evidence of disseminated MAI infection, investigation into an underlying immune defect was performed. Evaluation of acquired immunodeficiencies associated with MAI infections was done, with negative antibodies to HIV and HTLV 1 and 2, and so a search for a primary immunodeficiency with susceptibility to non-tuberculous mycobacteria ensued. Based on family history implying X-linked inheritance, NEMO deficiency seemed most likely. A single base change of the last base of exon 1b (c.1-16G>C) of IKKg resulting in abnormally spliced transcripts and reduced expression of NEMO was found. This datum confirmed the diagnosis of NEMO deficiency.

The patient was followed by the National Institutes of Health (NIH) clinic in Bethesda, Maryland. She was given gamma interferon and all the listed antibiotics and was improving along with tapering his corticosteroid use.

## Discussion

The transcription factor nuclear factor-kB (NF-kB) is a key transcription factor involved in regulating innate and adaptive immune responses as well as ectodermal development. NF-kB essential modulator (NEMO or inhibitor of NF-kB kinase gamma (IKKg)) is a 419 amino acid regulatory protein encoded by IKBKG on the X chromosome that is critical for NF-kB activation. In the resting state, NF-kB proteins are held by inhibitors of NF-kB (IkB) proteins. Activation of numerous cell receptors causes activation of the IkB kinase complex (IKK) including IKKa, IKKb, and IKKg or NEMO. The IKK complex then leads to phosphorylation of IKb proteins followed by ubiquitination thus freeing NF-kB to undergo nuclear translocation and activation of gene transcription.^10^

Amorphic NEMO mutations are lethal in boys, but hypomorphic mutations result in a combined immunodeficiency characterized by infectious susceptibility to bacterial, viral, pneumocystis, and mycobacterial infections starting in the first year of life. They can present with pneumonia, bacteremia, skin and soft tissue abscesses, enteritis or colitis, encephalitis or meningitis, sinusitis, or osteomyelitis.^9^,^11^

The immune phenotype of NEMO deficiency is variable but can include hypogammaglobulinemia with elevated IgM and IgA and reduced specific antibody responses due to impaired CD40-mediated B cell class switch recombination. TLR signaling and NK cell cytotoxicity are also diminished; T cell numbers and proliferation are variable.^9^,^11^

Due to perturbations in other pathways relying on NF-kB signaling, patients with NEMO deficiency may present with other manifestations including X-linked hypohydrotic ectodermal dysplasia, invasive pneumococcal disease and incontinentia pigmenti. While the majority of these diseases tend to present in childhood; exceptions are possible, and other primary immunodeficiency syndromes with defective innate and adaptive immunity may present later on in adulthood.

Hypomorphic mutations of the NEMO gene are responsible in the majority of patients who present with the rare NEMO deficiency syndrome.^12^,^13^ Ectodermal dysplasia (ED), one of the better characterized disorders resulting from deficiency of NEMO protein, shows the typical phenotype including thickened skin, conical teeth, absence of sweat glands, and thin, sparse hair.^14^ Similar to all NEMO syndrome variants, ED patients have a crippled innate immune system leading to a poor response to bacterial and fungal invasion, as well as difficulties in antibody production.^15^ Hypohydrotic ectodermal dysplasia with immune deficiency (HED-ID) results from an immunological aberration in the gene encoding the NF- κB essential modulator (NEMO; also known as IκB kinase γ subunit [IKKγ]).^16^ Classically, a hypomorphic coding mutation on the X-chromosome is responsible for the decreased function in NEMO, which leads to HED-ID.^4^ Yet, in 2010, Mooster et al. described a patient that had a normal coding sequence, but a securely deficient NEMO stemming from mutations in the noncoding region of the gene.^17^ Female carriers of this mutation develop anomalies of teeth, hair, skin, nails, and the CNS; however, the severity in female carriers depends mainly on X-chromosome lyonization, and thus is extremely variable.^16^ Male patients who have HED-ID suffer from reduced or absent sweat glands and hair follicles, dysgammaglobulinemia, and recurrent pyogenic infections of the integumentary, skeletal, and gastrointestinal systems.^16^ Also, due to lack of development of mucous glands and other anatomic abnormalities such as cleft palate, upper and lower respiratory tract infections are particularly prominent in these patients.^18^ In addition, atopic disease appears to be highly prevalent in HED-ID. In one series there was a 71% prevalence of eczema, 65% prevalence of asthma or recurrent wheezing, and 26% prevalence of food and drug allergies.^15^,^16^ This marked propensity of atopic disease is thought to be a result of impaired barrier function seen in the various organ systems affected by the ectodermal dysplasia syndromes.^18^

The vast majority of invasive pneumococcal disease (IPD) cases are unexplained, but in 2007 Ku et al described a child that had a hemizygous mutation in NEMO leading to a narrow clinical phenotype of susceptibility to *Streptococcus pneumoniae*.^19^ This patient had very mild signs consistent with anhydrotic ectodermal dysplasia, and from the age of 15 months was plagued several times with pneumococcal diseases.^19^ He developed buccal cellulitis and periorbital cellulitis, due to *S. pneumoniae* serotype 33.^19^ Despite being vaccinated with the heptavalent and 23-valent pneumococcal vaccinations, by the age of 2 years and seven months, he again developed blood and hip infections by *S. pneumoniae* serotype 23.^19^ The immunological profile of this patient showed that the antibody response to the immunizations was significantly blighted.^19^ In addition overwhelming infections with *Staphylococcus aureus*, *Pseudomonas* species and *Hemophilus influenza* have also been described. Susceptibility is also greatly variable among patients with some manifesting no infections to mild to very severe septic forms of disease.

Incontinentia pigmenti (IP), also known as Bloch-Sulzberger syndrome, is an X-linked dominant genodermatosis affecting primarily female newborns and is typically lethal in males.^20^ The estimated prevalence of IP is about 0.2/100,000 and results from a mutation in the NEMO gene leading to an inaccurate gene product and defective NF-κB activation.^21^ Even in patients with the same mutation, the phenotypic expression of this disease is highly variable. Due to the difficulty of diagnosis in mild cases, in 2013 Minic et al. proposed an updated version of the IP diagnostic criteria. There are four clinical stages IP patients progress through usually starting in the early neonatal period. These stages may occur concomitantly or sequentially and include the vesicular, verruciform, hyperpigmented, and hypopigmented stages.^21^ Linear vesicles, appearing within the first two months following birth, characterize the vesicular stage, to which follow verrucous hyperkeratotic plaques, thus indicating the verrucous stage. Brown to bluish-gray hyperpigmentation following Blaschko lines designates the third stage, which is followed by linear hypopigmented macules in the final stage^21^. The cutaneous findings are treated nonspecifically with topical steroids and emollients. However, in up to 80% of IP cases other extracutaneous clinical manifestations are present and comprise abnormalities of the teeth, eyes, hair, CNS, musculoskeletal systems, and the immune system.^21^ In June of 2014, Marques et al. published a case report highlighting the importance of early detection of IP. Seizures, ischemic strokes, strabismus, and cranial anomalies may result, and a multidisciplinary approach must be taken to address these extracutaneous signs.^20^

Santos et al., in 2006, uncovered an MSMD-causing NEMO mutation that curiously did not affect NF-κB activation. Rather, the mutations selectively impair CD40-triggered, and NF-κB/c-Rel mediated generation of IL-12 by monocytes, and thus explaining the specific susceptibility to mycobacteria.^4^ In their case reports, they describe a family with four maternally related males in successive generations with severe *Mycobacterium avium intracellulare* (MAI) infections. One of their patients was rather similar to our previous report and did not manifest disease until 13 years of age with extensive granulomatous cutaneous lesions initially thought to be sarcoidosis. These lesions turned out to be MAI of the skin.^4^ Other mutations in the IL-12/23-INF-γ circuit leading to MSMD, have been reported to result in infections with several other mycobacterial species, including *M. chelonae*, *M. fortuitum*, *M. smegmatis*, *M. kansasii*, and *M. szulgai*. However, to our knowledge, these species have not been found in cases related to mutations in NEMO, and we have not seen a report suggesting infection with *M. simiae*, like in our patient described previously. Its role as a pathogen in this case is very questionable. We cannot differentiate between infection and colonization, particularly if the lung is not involved. The presence of *M. Simiae* in the skin biopsies might also be related to the immune defect and was not reported previously in the NEMO deficiency patients.

## Conclusion

The occurrence of disseminated low-virulence atypical mycobacteria is rare in immunocompetent adults. When challenged with a patient such as the one we described, it is important to consider a range of genetic defects that may be causing this susceptibility. The primary considerations include IL-12/IFN-γ receptor defects resulting in MSMD, GATA2 deficiency, and acquired autoantibodies to IFN-γ, and highlights others that should be considered in the differential diagnosis **(**Table 1). Although NEMO deficiency typically manifests in childhood, our case demonstrates the phenotypic heterogeneity resulting from a genetic mutation in NEMO. Thus, clinical suspicion for defects in the NEMO gene should be high, and prompt genetic and immunological testing performed, when immunocompetent adults present with atypical mycobacterial infections.

## Figures and Tables

**Table 1:** Diseases That May Present in Adulthood with Susceptibility to Mycobacteria

Disease	Gene	Phenotype
GATA2 deficiency	GATA2	Dendritic Cell, Monocyte, B and NK Cell deficiency; Viral and Fungal Infections; Myelodysplasia, Leukemia, EBV associated smooth muscle tumor; Erythema nodosum, arthritis and lupus like syndrome^22^
WHIM Syndrome	CXCR4	Warts, Hypogammaglobuliemia, Recurrent bacterial Infections and retention of neutrophils in the bone marrow termed as Myelokathexis^23^
Anti-cytokine Autoantibody	Auto-antibodies targeted to IFN-γ and/or TNF-α	Common in East Asian population Recurrent Salmonella Infections Reactivation of latent varicella zoster^24^,^25^
NEMO Deficiency	NF-kB	Ectodermal dysplasia, invasive pneumococcal disease incontinentia pigmenti, Mendelian Susceptibility to mycobacterial diseases
X-linked Recessive Chronic Granulomatous Disease	CYBB	Isolated susceptibility to mycobacterial infections unlike the more common type of CGD^26^,^27^
Mendelian Susceptibility to	IL12B, IL12RB1,	Susceptibility to mycobacterial and viral infections; STAT1 deficiency is
Mycobacterial Diseases*	IFNGR1, IFNGR2, STAT1	generally mild compared to others^28^

* MSMD resulting from mutations in the IL-12/23 or IFN-γ receptors or STAT1 protein
